# Feasibility and Safety of Transjugular Liver Biopsy for Japanese Patients with Chronic Liver Diseases

**DOI:** 10.3390/diagnostics11010131

**Published:** 2021-01-16

**Authors:** Makoto Iijima, Takahiro Arisaka, Akira Yamamiya, Keiichi Tominaga, Kazunori Nagashima, Akira Kanamori, Satoshi Masuyama, Yuichi Majima, Kenichi Goda, Kazuyuki Ishida, Atsushi Irisawa

**Affiliations:** 1 Department of Gastroenterology, School of Medicine, Dokkyo Medical University, 880 Kitakobayashi Mibu, Tochigi 321-0293, Japan; mkiijima@dokkyomed.ac.jp (M.I.); aritaka@dokkyomed.ac.jp (T.A.); tominaga@dokkyomed.ac.jp (K.T.); n-kazu@dokkyomed.ac.jp (K.N.); k-akira@dokkyomed.ac.jp (A.K.); masu-526@dokkyomed.ac.jp (S.M.); y-majima@dokkyomed.ac.jp (Y.M.); goda@dokkyomed.ac.jp (K.G.); irisawa@dokkyomed.ac.jp (A.I.); 2 Department of Diagnostics Pathology, School of Medicine, Dokkyo Medical University, 880 Kitakobayashi Mibu, Tochigi 321-0293, Japan; ishida-k@dokkyomed.ac.jp

**Keywords:** transjugular liver biopsy, percutaneous liver biopsy, acute hepatic disease, chronic hepatic disease

## Abstract

Background and study aim: Transjugular liver biopsy (TJLB) can be used in patients who are ineligible for percutaneous liver biopsy (PLB) with acute and chronic hepatic disease. This study aimed to evaluate the usefulness and safety of TJLB in patients who were not indicated for PLB. Methods: Between July 2014 and February 2019, a total of 134 patients underwent liver biopsies at our institution. Among these, PLB was performed in 110 patients and TJLB in 24 patients. A retrospective comparison of clinical results in these patients was then performed. The primary endpoints of this study were the utility and safety of TJLB in patients who were not indicated for PLB. Results: The procedural success rate was 100% in both groups. The clinical response rate and the effective tissue sampling rate were 100% in the TJLB group and 97% in the PLB group (*p* = 0.55). There was no difference in the number of portal fields examined retrospectively between the two groups. No serious adverse events were observed in either group. Conclusions: It is suggested that TJLB is useful because it can be safely performed in patients with poor general condition who are not indicated for PLB.

## 1. Introduction

Liver biopsies are an important diagnostic method in acute and chronic hepatic disease. With biopsies, it is possible to examine the etiology, the degree of inflammation and fibrosis, and the responsiveness to treatment, making this method indispensable when searching for hepatic disease. In 1884, von Frerichs reported the percutaneous liver biopsy (PLB) procedure [1], after which various techniques, including ultrasound, computed tomography (CT) guidance, and laparoscopy have been performed for liver biopsies [2,3,4]. Because of its simplicity and utility, PLB is performed in numerous clinical settings as part of routine medical care. However, incidental adverse bleeding is sometimes an issue and may cause intra-abdominal hematoma or hemorrhagic ascites. In actuality, unexpected bleeding may occur even if the procedure is performed without technical issues. Moreover, the procedure is generally contraindicated in patients with accumulations of ascites and those who show bleeding tendencies such as abnormal coagulation; thus, pathological examination of the liver is difficult in such patients. Transjugular liver biopsy (TJLB) is recognized as an alternative liver biopsy procedure in patients contraindicated for PLB such as the aforementioned patients or those for whom bleeding is a concern. TJLB is a method for obtaining tissue by inserting a hard catheter for guidance into the hepatic vein through the jugular vein route. The procedure was reported in animal experiments using dogs by Dotter in 1964 [5] and was used in clinical application for the first time by Weiner and Hanafee in 1970 [6]. Because this procedure collects tissue from the intravascular space without penetrating the liver capsule, bleeding from the biopsy route and accumulations of ascites are not considered significant [3,7]. In other words, TJLB can be used in patients with abnormal coagulation, accumulations of ascites, acute hepatic failure, and those with excess fatty tissue or those who underwent a liver transplant and who are ineligible for PLB [8,9,10,11]. Furthermore, measuring the hepatic venous pressure gradient (HVPG) simultaneously is feasible with TJLB; thus, there is an advantage of being able to simultaneously diagnose portal hypertension and make a histological diagnosis in patients with hepatic cirrhosis. However, the procedure is more complicated than that of PLB, and thus is not commonly used. Moreover, because TJLB biopsy specimens have small and fragmented tissue, the quality of TJLB specimens has been reported as an issue compared to PLB. In this retrospective study, to evaluate the usefulness and safety of TJLB, we compared TJLB with PLB in patients in whom liver biopsies were performed at our institution. In particular, we evaluated the safety of TJLB in patients who were not indicated for PLB.

## 2. Materials and Methods

### 2.1. Study Design

This study is a retrospective cohort study conducted in a single institution that was approved by the ethics committee of Dokkyo Medical University Hospital. We provided a means to opt out instead of omitting informed consent, which was a way that guaranteed the opportunity for research subjects to opt out through a notification and publication of research information on our website. Between July 2014 and February 2019, a total of 134 patients underwent liver biopsies at our institution. Among these, PLB was performed in 110 patients and TJLB in 24 patients. A retrospective comparison of clinical results in these patients was then performed. The primary endpoints of this study were the utility and safety of TJLB in patients who were not indicated for PLB and the secondary endpoints were clinical response rate, tissue sampling rate, and rate of incidental adverse events with TJLB and PLB.

### 2.2. Indication for PLB and TJLB

TJLB was considered the first choice for patients in whom administration of antithrombotic drugs could not be interrupted during the biopsy procedure regardless of the drug or dose and patients with severe accumulations of ascites. Moreover, TJLB was indicated in patients with severe obesity in which percutaneous ultrasound-guided paracentesis was difficult. Of these, patients with prothrombin activity <20% and platelets <30,000/mm^3^ were, in principle, considered ineligible; however, even patients with prothrombin activity <20% and platelets >30,000/mm^3^ were subjected to the biopsy procedure after replacement of coagulation factors. PLB was indicated for all other patients.

### 2.3. PLB Procedure

The patients were asked to remain in the supine position with placement of their right hand behind the head in a hospital bed to avoid movement after the procedure and monitoring via pulse oximetry and of heart rate and blood pressure was performed. The point of puncture was marked by ultrasound imaging, usually between the sixth and eighth intercostal space in the right midaxillary line. Subsequently, the intercostal space was punctured 4 mm over the upper edge of the rib to avoid injury to the intercostal vasculoneurotic package. The puncture was performed with a 16G or 18G biopsy needle (Cook Medical, Bloomington, IN, USA). A dressing was placed over the incision and the procedure completed. Broad antibiotics were administered to all patients.

### 2.4. TJLB Procedure

TJLB was performed according to the standard technique using ultrasound and fluoroscopic guidance by experienced hepatologists [3]. Essential instruments for TJLB included: TJLB needle with a stiff cannula; long sheath 8 Fr; 0.035, Terumo guide wire; Amplatz super-stiff metallic guide wire; 5 Fr multipurpose catheter; and 18G intravenous cannula. The patient was placed in a supine position and an 8 Fr vascular access sheath was placed into the right internal jugular vein (IJV). A 5 Fr multipurpose catheter (Cook Medical, Bloomington, IN, USA) with an angled tip Terumo (Terumo, Tokyo, Japan) guide wire was navigated via the sheath across the inferior vena cava, and into the right hepatic vein (RHV), and venograms were taken via the multipurpose catheter to ensure its position in the RHV. A 260 cm, Amplatz super-stiff guide wire (Cook Medical) was placed into the RHV through the 5 Fr catheter. Subsequently, the 7 Fr angled metallic cannula of the TJLB set (Cook Medical) was passed over the Amplatz super-stiff guide wire into the RHV and set in the appropriate position in the RHV. Thereafter, the biopsy needle was fixed at an appropriate position thus ensuring the depth and angle. After confirmation of the appropriateness of the position using a cone beam CT, a 60 cm long, 18G, loaded biopsy gun (Quickcore, Cook Medical) was passed through the metallic cannula and the liver was punctured through the wall of the RHV. Basically, the puncture was performed twice or more to obtain adequate/sufficient samples. The obtained samples were immersed in formalin. If samples were adequate, a check venogram was performed to rule out any active contrast leaks. After these procedures, the TJLB cannula was then removed and the IJV access site was compressed for 10 min.

### 2.5. Assessment of Adverse Events

Postprocedural vital signs including oxygen saturation were monitored, initially every 30 min for 2 h followed by every 6 h for 24 h. Minor and major adverse events were classified according to the Society of Interventional Radiology criteria [12]. Patients suspected of experiencing adverse events were closely monitored and subjected to serial testing with hemoglobin, hematocrit, and blood chemistry.

### 2.6. Histrogical Evaluation/ Definition of Adequate for Reporting

The specimens without portal fields were ineligible. Adequate for reporting was defined as specimens that included portal fields and were judged by the pathologist to be sufficient for diagnosis. In addition, for the cases that could be evaluated retrospectively, we again counted the number of portal areas and evaluated the diagnostic ability of TJLB and PLB.

### 2.7. Statistical Analysis

Continuous variables were reported as means ± standard deviation (SD), whereas categorical data were expressed as frequencies. The Mann–Whitney U test was used to compare the differences in continuous variables, the Pearson χ^2^ test or Fisher exact test was used to compare categorical variables between the 2 groups. *p* < 0.05 was considered statistically significant. All statistical analyses were performed using SPSS software version 21.0 (International Business Machines Co., Tokyo, Japan).

## 3. Results

### 3.1. Indication for TJLB

The background of reasons why TJLB was selected instead of PLB is shown in Table 1. The most common reason why TJLB was selected was due to ascites accumulation in 17 patients (71%). Next, abnormal coagulation was observed in 3 patients (13%), low platelets in 2 patients (8%), and continued administration of antithrombotic drugs during the liver biopsy was needed in 1 patient (0.4%). The present study did not include patients for whom PLB would be difficult due to obesity.

### 3.2. Underlying Liver Disease

The background diseases that resulted in patients needing liver biopsies are listed in Table 2. Among the 24 patients who underwent TJLB, 7 patients had a main background disease of autoimmune hepatitis (AIH), 4 patients had drug-induced liver injury, 3 patients had alcoholic hepatopathy, and 2 patients had non-alcoholic steatohepatitis (NASH). Conversely, among the 110 patients who underwent PLB, the main background diseases were AIH in 27, hepatocellular carcinoma (HCC) in 17, intrahepatic cholangiocarcinoma (ICC) in 8, metastatic liver tumors in 8, NASH in 7, and congested liver in 6 patients.

### 3.3. Characteristics of Patients Who Underwent TJLB and PLB

Patient demographics are shown in Table 3. The mean age was 55 years in the TJLB group and 59 years in the PLB group, and sex was male in 50% (12/24) in the TJLB group and 52% (57/110) in the PLB group, showing no significant difference between the groups. However, international normalized ratio(PT-INR) was 1.5 in the TJLB group and 1.1 in the PLB group, and PT-INR was significantly prolonged in the TJLB group (*p* < 0.001). Platelets were 14.0 × 10^3^/μL in the TJLB group and 21.5 × 10^3^/μL in the PLB group, showing a significant decrease in platelets in the TJLB group (*p* < 0.001). Bilirubin was 7.6 mg/dL in the TJLB group and 2.6 mg/dL in the PLB group, showing a significant increase in bilirubin in the TJLB group (*p* < 0.001). The Child–Pugh score was 9.9 in the TJLB group and 6.4 in the PLB group, showing a significantly higher score in the TJLB group (*p* < 0.001). The rate of regular use of antithrombotic drugs, weight, aspartate transaminase (AST), and alanine transaminase (ALT) did not significantly differ between the two groups.

### 3.4. Outcomes of TJLB and PLB

The results of TJLB and PLB procedures are shown in Table 4. The number of punctures was 2.4 in the TJLB group and 1.4 in the PLB group, showing a significantly higher number in the TJLB group (*p* < 0.001). The procedural success rate was 100% in both groups. The clinical response rate and the effective tissue sampling rate were 100% in the TJLB group and 97% in the PLB group, showing high results with no significant difference between the two groups. A minor adverse event was observed in one patient in the TJLB group, but no serious adverse events were observed in either group.

### 3.5. Number of Portal Fields

For the cases that could be evaluated retrospectively, the results of number of portal fields are shown in Table 5. The number of portal fields was confirmed by medical reports in 24 cases of TJLB and 46 cases of PLB. The number of total portal fields was 12.5 in the TJLB group and 7.8 in the PLB group, showing high results with no significant difference between the two groups. The number of average portal fields was 5.7 in the TJLB group and 6.8 in the PLB group, showing high results with no significant difference between the two groups.

## 4. Discussion

In the current study, we evaluated the feasibility and safety of TJLB performed in high-risk patients. The results showed that TJLB can be performed safely and reliably in patients with severe ascites accumulation or severe coagulation abnormalities and patients who require continuous administration of antithrombotic drugs who are not indicated for PLB. Furthermore, although PLB was associated with a higher number of punctures, the specimen amounts obtained were also sufficient for pathological diagnosis, and the procedure was performed safely without any serious incidental adverse events. Our data demonstrated the safety and utility of TJLB in patients not indicated for PLB.

Liver biopsies are generally performed percutaneously, mainly for the diagnosis of chronic hepatitis and liver tumors. Ultrasound-guided PLB, which is a safe, simple, and reliable biopsy method, has been established as a basic procedure for PLB [2,13,14]. Furthermore, in patients with severe obesity in whom it is difficult to visualize the liver on ultrasound, PLB can be technically difficult to perform and endoscopic ultrasound guided liver biopsy (EUS-LB) may be selected [15,16]. However, in patients with accumulations of ascites, hepatic failure, blood dyscrasia, or those with severe coagulation abnormalities due to oral drugs or other reasons showing bleeding tendencies, PLB or EUS-LB is more likely to cause prolonged intra-abdominal bleeding and, since there are no non-invasive measures to stop the bleeding, these patients are in general contraindicated. McCaty et al. [16] performed a systematic review and meta-analysis and reported that EUS-LB appears to be a safe and minimally invasive procedure that is comparable to PLB and TJLB in terms of biopsy specimens obtained and rate of adverse events associated with each method. However, in this study, a sub-analysis on the safety in patients with coagulopathy/taking anticoagulant agents was not performed. In general, EUS-guided fine-needle aspiration (EUS-FNA) for various diseases should be performed with caution in patients with abnormal coagulation [17], and the recommendation is to consider alternative biopsy methods if possible. With TJLB, safely collecting liver tissue is feasible even in the aforementioned high risk patients who are ineligible for PLB under ultrasound guidance or in patients in whom the procedure is difficult to perform, and patients benefit from the increase in techniques to choose from [18,19,20,21,22].

In terms of incidental adverse events, previous reports to date have stated frequencies of 1.3–20% for TJLB [2,3,9,23]. Mild incidental adverse events such as pyrexia, tachycardia, hematoma, and serious incidental adverse events such as pneumothorax, cervical pseudoaneurysm, biliary hemorrhage, and intra-abdominal bleeding have been reported. As large-scale data, Kalambokis et al. performed a systematic review of TJLB from 64 articles in 7649 patients and reported that the incidence of mild and severe complications was 6.5% and 0.56%, respectively [8]. In our study, no mild or severe incidental adverse events were reported, showing lower frequencies than those in previous reports. In addition, in the TJLB group, there were patients in whom the administration of the antithrombotic drugs could not be interrupted and the patients were continuously treated with the drugs during the procedure. However, there were no incidental adverse events and the procedures could be performed safely. Although not reported in our study, intra-abdominal bleeding is known to be a very rare complication of TJLB. The mechanism of onset of intra-abdominal bleeding in TJLB is as follows. During paracentesis, if the puncture needle penetrates the liver capsule, then the risk of intra-abdominal bleeding increases. In a previous report, 0.59% of patients undergoing TJLB experienced intra-abdominal bleeding [18] and caution should be exercised since this procedure is performed in patients with coagulation abnormalities, which may lead to serious complications. As a countermeasure, if intra-abdominal bleeding is suspected, post-TJLB contrast-enhanced imaging examinations are recommended [24].

The first documented successful specimen collection with TJLB was reported by Dotter in dogs in 1964 [25]. The number of punctures was 2.4 in the TJLB group, which was significantly higher than that with the PLB group in this study. The number of punctures was also reported to be an average of 2.7 punctures in the systematic review by Kalambokis et al. [8], which was similar to our results. For TJLB, the ideal number of biopsies is considered to be three or four when using a 19G Tru-Cut-type biopsy needle and, if a specimen with a total length of at least 30 mm can be collected, sufficient pathological evaluation is reportedly feasible [26]. In patients with chronic hepatitis C and NASH undergoing liver biopsies, obtaining a tissue length of 20–25 mm or at least 11 portal regions is reportedly necessary to reduce sampling error and to accurately diagnose the severity of hepatitis [27,28]. Cholongitas et al. reported that it is difficult to obtain at least 11 portal regions without performing at least three punctures, and that only 60% of tissues that are at least 28 mm in length contain at least 11 portal regions [29].

In humans, transjugular percutaneous cholangiography was reported by Hanafee et al. in 1967 [30] and the first clinical application of TJLB was reported by Weiner et al. in 1970 [6]. Thereafter, Rösch et al. [31] conducted full-fledged clinical studies in 1973 and numerous reports have since been published. TJLB is a method of collecting tissue by puncturing the liver parenchyma from the hepatic vein side with a biopsy needle via a catheter placed in the hepatic vein. In recent years, with improvement of puncture needles and paracentesis techniques, numerous specimens can be obtained in a single procedure [32,33]. Although TJLB is a safe procedure, it has the potential to be invasive and there are potential complications, and it is not well accepted by patients. Accelerated noninvasive testing to diagnose and stage chronic hepatitis is needed; however, there are still no suitable complete substitutes for liver biopsies. Thus, liver biopsies remain an important diagnostic procedure in clinical practice, for example, to differentiate simple steatosis from hepatic steatosis in patients with nonalcoholic fatty liver disease (NAFLD) or to obtain diagnostic and prognostic information on autoimmune hepatitis and alcoholic hepatitis [34]. TJLB is recognized as an established procedure in the United States and Europe, and it gives important information in various pathological conditions. However, there have been few reports on the utility of TJLB in Japan and, since it is a complicated procedure, it has not become widespread. This study is important in that it provides data reflecting the actual status of use of TJLB in Japan.

Limitations of this study include that it is a single-center retrospective study, that the number of patients is small, and that there are few outcome events. Moreover, it was difficult to evaluate the quality of the specimens in all cases, such as specimen length and portal fields because of retrospective study. However, we were able to examine the number of punctures and portal fields in a total of 70 patients, and found that there was no difference in the number of punctures and portal fields between the TJLB and PLB groups. In other words, TJLB may have the same diagnostic ability as PLB.

## 5. Conclusions

It is suggested that TJLB is useful because it can be safely performed in patients with poor general condition who are not indicated for PLB. Although TJLB was developed a long time ago, with the advent of an aging society in Japan, the number of patients receiving antithrombotic drugs centering on the field of heart disorders and cerebrovascular disease will steadily increase, and the need for the procedure will also increase. Thus, the present results need to be validated in prospective and large-scale clinical studies.

## Figures and Tables

**Table 1:** Indication for transjugular liver biopsy (TJLB).

Indication	Frequency, *n* (%)
Coagulopathy	3 (12.5)
Low platelets	2 (8.3)
Anticoagulant drug	1 (0.4)
Difficulty of percutaneous liver biopsy (PLB)	0 (0)
Patient’s factors	
Ascites	17 (70.8)
Obesity	0 (0)
Others	1 (0.4)
Factors of the procedure	0 (0)
Refusal of PLB	0 (0)

**Table 2:** Underlying liver diseases.

Indication of biopsy	TJLB (*n* = 24)	PLB (*n* = 110)
Hepatitis B	0	0
Hepatitis C	0	2
Hepatocellular carcinoma	0	17
Cholangio cellular carcinoma	0	8
Metastatic liver tumor	0	8
Alcoholic liver disease	3	0
Nonalcoholic steatohepatitis	2	7
Autoimmune hepatitis	7	27
Primary sclerosing cholangitis	1	4
Primary biliary cirrhosis	0	3
Congestive hepatopathy	1	6
Hepatic failure	1	1
Drug-induced liver injury	4	5
Cryptogenic cirrhosis	1	4
Graft-versus-host disease	1	4
Portal hypertention	1	1
Hemochromatosis	1	1
Malignant lymphoma	0	1
Others	1	11

**Table 3:** Characteristics of patients who underwent TJLB and PLB.

Parameters	TJLB (*n* = 24)	PLB (*n* = 110)	*p* Value
Age (Years)	55.2 ± 17.4	55.2 ± 17.4	0.39
Male:Female	12:12	57:53	0.87
Weight, kg	58.4 ± 9.8	59.2 ± 14.0	0.92
International normalized ratio (PT-INR)	1.5 ± 0.4	1.1 ± 0.1	<0.001
Platelets, ^10^3^/μL	14.0 ± 9.7	21.5 ± 10.3	<0.001
Alanine transaminase (ALT) U/L	118.8 ± 201.7	126.6 ± 203.2	0.46
Aspartate transaminase (AST) U/L	141.5 ± 227.1	105.1 ± 154.7	0.34
Bilirubin, mg/dL	7.6 ± 6.7	2.6 ± 5.1	<0.001
Child–Pugh score	9.9 ± 1.6	6.4 ± 1.4	<0.001
Anticoagulant drug, *n* (%)	3 (12.5)	11 (10.0)	0.48

**Table 4:** Outcomes of TJLB and PLB.

Parameters	TJLB (*n* = 24)	PLB (*n* = 110)	*p* Value
Number of punctures, *n*	2.4 ± 0.8	1.4 ± 0.6	<0.001
Success rate of technique, *n* (%)	24 (100)	110 (100)	
Clinical success, *n* (%)	24 (100)	107 (97.2)	0.55
Adequate for reporting, *n* (%)	24 (100)	107 (97.2)	0.55
Complications overall, *n* (%)	1 (4.2)	0 (0)	0.18
Major adverse events *, *n* (%)	0 (0)	0 (0)	
Minor adverse events **, n (%)	1 (4.2)	0 (0)	0.18

* Major adverse events: Adverse events prolonged the hospitalization. ** Minor adverse events: Adverse events didn’t prolog the hospitalization.

**Table 5:** Number of portal fields.

Parameters	TJLB (*n* = 24)	PLB (*n* = 110)	*p* Value
Number of total fields, *n*	12.5 ± 4.1	7.8 ± 2.1	0.61
Number of punctures, *n*	2.4 ± 0.8	1.2 ± 0.4	0.70
Number of average portal fields, *n*	5.7 ± 2.3	6.8 ± 2.3	0.22

## Data Availability

No new data were created or analyzed in this study. Data sharing is not applicable to this article.
